# Mitochondrial Analysis of the Most Basal Canid Reveals Deep Divergence between Eastern and Western North American Gray Foxes (*Urocyon* spp.) and Ancient Roots in Pleistocene California

**DOI:** 10.1371/journal.pone.0136329

**Published:** 2015-08-19

**Authors:** Natalie S. Goddard, Mark J. Statham, Benjamin N. Sacks

**Affiliations:** 1 Mammalian Ecology and Conservation Unit, Veterinary Genetics Laboratory, School of Veterinary Medicine, University of California-Davis, Davis, California, United States of America; 2 Department of Population Health and Reproduction, School of Veterinary Medicine, University of California-Davis, Davis, California, United States of America; BiK-F Biodiversity and Climate Research Center, GERMANY

## Abstract

Pleistocene aridification in central North America caused many temperate forest-associated vertebrates to split into eastern and western lineages. Such divisions can be cryptic when Holocene expansions have closed the gaps between once-disjunct ranges or when local morphological variation obscures deeper regional divergences. We investigated such cryptic divergence in the gray fox (*Urocyon cinereoargenteus*), the most basal extant canid in the world. We also investigated the phylogeography of this species and its diminutive relative, the island fox (*U*. *littoralis*), in California. The California Floristic Province was a significant source of Pleistocene diversification for a wide range of taxa and, we hypothesized, for the gray fox as well. Alternatively, gray foxes in California potentially reflected a recent Holocene expansion from further south. We sequenced mitochondrial DNA from 169 gray foxes from the southeastern and southwestern United States and 11 island foxes from three of the Channel Islands. We estimated a 1.3% sequence divergence in the cytochrome *b* gene between eastern and western foxes and used coalescent simulations to date the divergence to approximately 500,000 years before present (YBP), which is comparable to that between recognized sister species within the Canidae. Gray fox samples collected from throughout California exhibited high haplotype diversity, phylogeographic structure, and genetic signatures of a late-Holocene population decline. Bayesian skyline analysis also indicated an earlier population increase dating to the early Wisconsin glaciation (~70,000 YBP) and a root height extending back to the previous interglacial (~100,000 YBP). Together these findings support California’s role as a long-term Pleistocene refugium for western *Urocyon*. Lastly, based both on our results and re-interpretation of those of another study, we conclude that island foxes of the Channel Islands trace their origins to at least 3 distinct female founders from the mainland rather than to a single matriline, as previously suggested.

## Introduction

Zoological systematics has historically relied on morphological variation to designate taxa. More recently, molecular data have revealed many cases where highly divergent species were not recognized as such [1]. These findings, in turn, greatly enhance our ability to understand evolutionary processes by visualizing better the texture of evolutionary products. In particular, vertebrate populations of North America are frequently split into eastern and western lineages that diverged in isolation during past glacial periods [2]. This general phylogeographic pattern, which has many variations, has been confirmed or discovered in several mammalian carnivores using mitochondrial sequences. More ancient splits that predate the Illinoian (penultimate) glaciation (190–130 KYA) typically have resulted in distinct sister species, such as the swift fox (*Vulpes velox*) and kit fox (*Vulpes macrotis*), and American marten (*Martes americana*) and Pacific marten (*M*. *caurina*) [3,4]. Other, more recent splits, tracing to the late Illinoian or Wisconsin glaciation, include bobcats (*Lynx rufus*) and red foxes (*Vulpes vulpes*) [5–6]. Secondary contact between long-separated lineages can be a source of novel gene combinations that can fuel future evolution, particularly when refugial populations evolved in distinct environments or were separated for long periods of time [7]. Thus, estimating divergence times and identifying Pleistocene refugia have important implications for understanding evolutionary distinctiveness and potential of contemporary populations.

In the present study, we sought to improve our understanding of the continent-wide phylogeography of the gray fox (*Urocyon cinereoargenteus*) and its congener, the island fox (*U*. *littoralis*), which together represent the most basal clade within the extant Canidae [8,9]. The lineage leading to *Urocyon* diverged from other canids during the Miocene, 8–12 million years (MY) ago [10]. Fossil *Urocyon* has been present in North America since the early Pliocene (~5 MY) and contemporary *Urocyon* represents the only extant canid with a natural range spanning both North and South American continents [11]. Contemporary gray foxes have been classified into 15 subspecies based on morphology (in additional to 6 subspecies of island fox) [12]. However, high local variability can also serve to obscure deeper evolutionary distinctions. Thus, depending on range dynamics throughout the past 2 million years of climatic fluctuations of the Quaternary period, very ancient splits within this genus seem likely, and should be most evident between eastern and western extents at the northern end of the range.

Second, we were interested in evaluating the role of the California Floristic Province (hereafter, California) as a Pleistocene refugium for western *Urocyon*. California served as an important Pleistocene refugium for many species and is among the most biodiversity-rich regions on earth [13–15]. Because of its high diversity of habitat and microclimate, California is thought to have been an important engine of evolutionary diversification on multiple time scales [13]. Many endemic California vertebrates, including those in the northern portion of the state, trace their ancestry in-situ several million years into the past [16] and even vagile carnivores, such as the coyote (*Canis latrans*), are thought to trace their California ancestry at least well into the Pleistocene if not further back in time [11,15]. Thus, we hypothesized that contemporary California gray foxes also reflect a long-standing lineage that diversified in-situ.

Alternatively, recent evidence suggests the possibility of a much more fluid scenario whereby western gray fox expanded and contracted their ranges over hundreds of kilometers in response to climatic fluctuations in California. In particular, Hofman et al. [17] found island foxes inhabiting the Channel Islands off southern California to share a more recent common ancestral matriline with gray foxes sampled in northern than in southern California, leading them to hypothesize that western gray foxes shifted their ranges significantly southward around the last glacial maximum and northward during the Holocene in response to shifting habitats [17]. Because that study’s focus was on the island foxes, only a small number (*n* = 25) of gray foxes was sequenced, which precluded an in-depth analysis of phylogeography in mainland gray foxes. However, both hypotheses entail several predictions that can be readily tested with a larger sample. First, if gray foxes in northern California were established (or significantly augmented) through a recent expansion, we would expect to find low genetic diversity and low geographic structuring among matrilines (e.g., [18–20]). Second, we should observe characteristic signatures of demographic expansion in terms of the haplotype and nucleotide diversities [5,19,20,21]. Conversely, if northern California gray foxes reflect long-standing in-situ evolution, we predict relatively high diversity and localization of recently derived subclades and no signal of expansion [15,18,22]. Additionally, a decline in population size could potentially explain the apparent loss (or reduced frequency) of island fox-like haplotypes from southern California gray foxes, as population declines accelerate loss of matrilines through random genetic drift.

**Fig 1:**
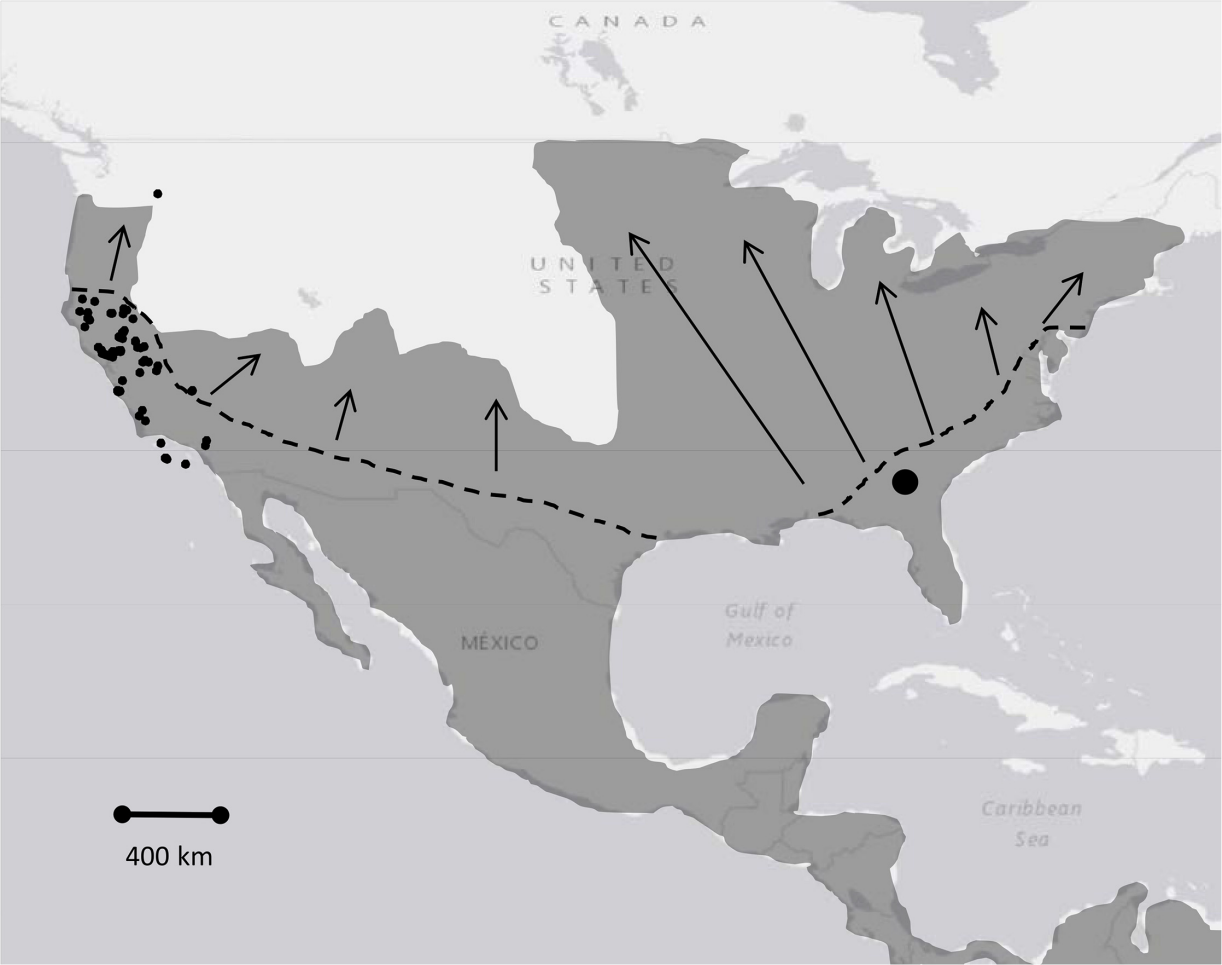
Approximate range of the gray fox (*Urocyon cinereoargenteus*). Dashed line indicates hypothetical northern boundary at the last glacial maximum and arrows denote hypothetical post-Pleistocene expansion [11]. Filled circles represent approximate locations of gray and island (*U*. *littoralis*) foxes used in the present study from the southwestern (*n* = 131 gray fox; 11 island fox) and southeastern (*n* = 38) portions of the range. Southeastern samples were from a 300 km^2^ region, represented by a single large filled circle.

Our first objective was to estimate the divergence separating eastern and western gray (and island) foxes. Although gray foxes today occur at least as far north as Canada in the east and northern Oregon in the West, we focused our sampling in the southeast (Georgia) and southwest (California), which were either shown [20] or hypothesized [15] to represent Pleistocene refugia for gray foxes and other temperate carnivores (Fig 1). Our second objective was to test the latter hypothesis and investigate the phylogeography and historical demography of *Urocyon* in California, including to re-evaluate the timeline for diversification of western *Urocyon* presented previously [17].

## Materials and Methods

### Samples

For this study we used 169 gray fox and 11 island fox samples collected during 1998–2014 from throughout California and vicinity (*n* = 141) and Putnam County, Georgia (*n* = 38), as well as a sample from Washington state (Yakima County); to our knowledge, this was the first record of gray fox north of the Columbia River (Fig 1). Gray fox samples from California (or just outside) were distributed as follows: Central Coast (*n* = 10), Western Cascades (*n* = 16), North Coast (*n* = 35), Southern California (*n* = 2), Western Sierra Nevada/San Joaquin Valley (*n* = 14), Sacramento Valley (*n* = 32), and the Eastern Sierra Nevada (*n* = 12). We also sampled 11 island foxes from Santa Cruz (*n* = 4), San Nicholas (*n* = 5), and San Clemente (*n* = 2) Islands. Samples included 40 whole blood, 26 tissue, 5 hair, and 109 scat samples. The noninvasive samples (hair, scats) were drawn from an archive of DNA extracts from scats collected by trained students or staff as part of many unrelated studies targeting various mesocarnivores (e.g., [23, 24]); in all cases, scat sample preservation involved submersion of ~2 g of scat in >4 times that volume of 95–100% ethanol. All archived noninvasive DNA samples had been categorized to species via mitochondrial sequencing as described previously (e.g., [23, 24]) prior to inclusion in this study. No animals were harmed for this study and all samples were covered by appropriate permits and permissions. All field collection procedures were approved by the University of California, Davis, Animal Care and Use Committee (IACUC No. 17860) and the California Department of Fish and Wildlife through scientific collecting permits and memoranda of understanding. Permission also was secured for all field collections on private and public lands. Field collections did not involve endangered or protected species. Blood samples were collected in the field by B. N. Sacks via venipuncture after cage trapping and immobilization with ketamine HCl (10 mg/kg) and xylazine HCl (2 mg/kg); all foxes were released unharmed. We also used samples that had been previously deposited in institutional collections at the University of California, Davis Veterinary Medical Teaching Hospital and Mammalian Ecology and Conservation Unit, covered by the appropriate state and federal permits.

### Laboratory Procedures

To avoid contamination of noninvasive samples with DNA from tissue samples, laboratory procedures were performed for noninvasive samples in a separate dedicated space. We extracted DNA using Qiagen DNeasy blood and tissue kits (Qiagen Inc.) for blood and tissue samples and QIAamp DNA stool kits for scats, following manufacturer’s protocols. For hair extractions we digested the follicles overnight, followed by DNA purification using phenol/chloroform, and cleaned up using Amicon Ultra centrifuge filters (Millipore ltd; [24]).

We performed polymerase chain reaction (PCR) to amplify a 441 bp (including primers) portion of mitochondrial cytochrome *b* using primers (RF14724, RF15149; [25]), PCR chemistry, and cycling conditions described by Aubry et al. [5]. After trimming primers and an additional 34 bp from the 3’ end that could not be consistently sequenced from the forward direction, we obtained a 363 bp fragment. We also performed PCR to amplify a portion of D loop using the same primers (H16498 and L15910; [26]) and thermoprofile used by Bozarth et al. [20] and the PCR chemistry described by Aubry et al. [5]. After primer trimming, we reduced this 428–432 bp fragment to 422 bp by excising a 4–10-bp cytosine homopolymer that was highly variable and of ambiguous alignment. We also used a second trimming to the 406 bp used by Bozarth et al. [20] to facilitate direct comparisons to that data set of 229 gray foxes from the southeastern and northeastern United States. (Although Bozarth et al. [20] reported using a 411 bp fragment, 5 bp of primer sequence apparently were included on the 3’ end.)

We sequenced fragments as previously described [19] in both directions for D loop and only in the forward direction for cytochrome *b*. We did not deem it necessary to sequence cytochrome *b* in the reverse direction as we observed high consistency and base-quality in the first 363 bp and a low frequency of novel substitutions.

We used Basic Local Alignment Search Tool (BLAST) to verify novel sequences against haplotypes accessioned in the nucleotide database in GenBank [27], and additionally compared D loop sequences manually to those reported by Bozarth et al. [20], prior to depositing novel sequences in GenBank. We estimated haplotype and nucleotide diversity using Arlequin 3.5 [28].

We based analyses on our sample using both the 363 bp portion of cytochrome *b* and the 422 base-pair portion of D loop. We concatenated these linked mtDNA sequences into a 785 bp fragment.

### Data Analysis

We constructed phylogenetic trees from the 785 bp haplotypes using both maximum likelihood and Bayesian approaches with trees rooted to red fox and coyote outgroups (GenBank Nos. AB292747, KF661096). The most appropriate DNA substitution model (HKY [29]) was determined in MEGA 6.06 [30] and used to construct maximum likelihood trees with bootstrap support evaluated based on 500 replicates in MEGA 6.06. For the Bayesian tree, we partitioned the cytochrome *b* character set by codon position. We used the HKY DNA substitution model for cytochrome *b* and the HKY+Γ model for D loop, as determined by jModelTest (0.1.1; [31]). We computed Bayesian trees using MrBayes 3.1.2 [32], running the model for 10^7^ generations, with four MCMC simulations running simultaneously. Sampling included one tree every 1,000 generations. Based on inspection of MrBayes output trace values using Tracer v1.5 (Rambaut A, Drummond AJ, 2007, http://beast.bio.ed.ac.uk/Tracer), we determined that convergence occurred during the first 10% of generations, which we discarded as burn-in.

To convert scaled estimates to time, we assumed the same mutation rates as previously for red and eastern gray foxes, specifically 2.8% per million generations for cytochrome *b* and 17.75% per million generations for D loop [5,20]. Averaging these estimates (weighted by sequence length) resulted in an estimated mutation rate for the total 785 base pair sequence of 10.8% per million generations or 8.51 x 10^−5^ mutations per generation. Although these previous studies have based divergence times in foxes conservatively on a generation time of 1 year, this is the absolute minimum theoretically possible for a monestrous canid (and which would imply semelparity!). Therefore, we present estimates in terms of generations to enable direct comparisons with these studies, but also interpret estimates in terms of a more realistic generation time. To obtain such an estimate, we utilized a dataset for the ages of 435 gray foxes from the southeastern US [33]. We regressed the natural logarithm of the number of individuals in each adult year class on year class, constraining the intercept to zero, and then exponentiated the estimated slope to produce the annual adult survival estimate. We then estimated from this distribution the average adult age at 1.72 years [34]. Because fecundity tends to be slightly higher in older canids (e.g., [35]), the generation time (i.e., average age of all newborns’ mothers) was likely closer to 2 years.

To estimate the divergence time separating eastern and western *Urocyon*, we used MCMC simulations in the program IMa2 [36]. We used the HKY model of DNA substitution and the generational substitution rate indicated above to run our analysis. We performed multiple initial runs parameterized to allow for migration (isolation with migration model), but migration values consistently approached zero. Therefore, we only report results for the isolation-only model, which best described the data. We completed multiple short runs in MCMC mode to evaluate mixing and narrow the parameter space. We then ran five independent runs (different random number seeds) of 2 x 10^6^ steps in MCMC mode, with a burn-in of 250,000 to confirm consistency. We estimated joint distributions and computed final parameter estimates and 95% highest posterior densities (HPD) using “LoadTree” mode.

To assess the historical demography of western *Urocyon*, we tested for population growth by calculating Fu’s *F*_S_ [21] and comparing to a distribution of the same statistic calculated from coalescent simulations under from null expectations (i.e., no population growth) in DnaSP 4.50 [37]. We then constructed a Bayesian skyline plot, a nonparametric Bayesian coalescent-based approach utilizing MCMC sampling to estimate the posterior distribution of effective population size through time, in the program BEAST 1.75 [38,39]. We used a HKY substitution model for cytochrome *b* (partitioned into first, second, and third positions) and a HKY+ Γ for the D loop, with 10 skyline groups, and a strict molecular clock. We assumed a mutation rate of 10.8% per million generations (as above). We ran 100 million cycles sampling every 10,000 cycles. We visualized the results in Tracer 1.5 (provided with BEAST). We combined the results of two independent runs (ESS values >200) and discarded the first 10% as burn-in using the program Logcombiner 1.75 (provided with BEAST).

For genetic structure analyses in the western foxes, we visualized the relationships among western haplotypes using a median-joining network [40] produced within Network (v 4.613). To interpret patterns with respect to ancestor-descendent relationships, we used the eastern gray foxes and outgroups (coyote, red fox) to root the network. However, to avoid having to literally interpret a particular rooting point with low statistical support, we relied primarily on “tip” versus “interior” placement on the network to assess polarity [22]. We estimated time to most recent common ancestor of nested clades (or haplogroups) based on the average number of mutations (rho) separating ancestral and descendent haplotypes and converted to time estimates assuming the same mutation rate indicated above [41,42]. We used SAMOVA (2.0) to evaluate population structure using Φ_ST_ based on pairwise differences [43–45]. We ran 100 iterations at each number of geographical groupings (*K*) ranging from 2 to 7. We evaluated isolation by distance using Mantel tests between geographical distances and pairwise Φ_ST_ values computed in Arlequin 3.5 with 1,000 permutations.

Using 406 bp of D loop sequence common to our study and that of Bozarth et al. [20], we pooled samples to construct an unrooted maximum likelihood tree of all known eastern and western *Urocyon* haplotypes. We then separated our 406 bp D loop sequences into samples of southeastern gray foxes, mainland western gray foxes, and island foxes, as well as regional subsamples of the western gray foxes, and estimated haplotype and nucleotide diversity for direct comparison to the southeastern gray foxes and northeastern gray foxes (known to reflect a Holocene expansion) analyzed by Bozarth et al. [20].

We compared our findings to those based on the mitogenome data set of Hofman et al. [17] (GenBank accession numbers KP128924- KP129108, NC_026723) as follows. First, we aligned their 185 western *Urocyon* (and one eastern gray fox) sequences to our 785 bp data set to retrieve the orthologous bases, which we combined with our concatenated sequences. We then constructed a network from this combined dataset to enable a direct comparison of haplotypes between studies. Next, we generated a Bayesian skyline plot using the whole mitogenomes from the 26 western mainland gray foxes of Hofman et al [17]. We also generated a Bayesian skyline plot combining our concatenated (785-bp) sequences with those of the 26 western gray foxes of Hofman et al. [17], but found no qualitative difference from the one estimated solely from our data and therefore did not report results here. Lastly, we re-constructed a network composed of the 185 whole mitogenomes of Hofman et al. [17] (i.e., essentially identical to theirs) from which we calculated rho estimates (as described above) for comparison to time estimates based on our 785 bp sequences.

## Results

We obtained 180 cytochrome *b* sequences representing 13 distinct haplotypes (GenBank Accession Nos. KP888884–KP888896), 8 of which had been previously observed in western *Urocyon* [17]. Haplotype diversity was slightly higher in the west (0.70, SD = 0.032) than east (0.63, SD = 0.063), but nucleotide diversity was similar between the west (0.0028, SD = 0.00023) and east (0.0030, SD = 0.00044).

We also sequenced 145 of these samples at the D loop fragment, producing 25 haplotypes, 16 of which were novel. Otherwise, 7 of our 19 western *Urocyon* haplotypes [17] and 2 of our 6 eastern *Urocyon* haplotypes [20] had been previously reported. We deposited all 19 western and the 4 novel eastern haplotypes named in this study in GenBank (Accession Nos. KP888858–KP888879), retaining the names provided by Bozarth et al. [20] for the two previously reported eastern haplotypes (Uci 22, Uci 23).

We produced full concatenated (785 bp) sequences for the 145 foxes, which, unless otherwise mentioned, were used for subsequent analyses (Table 1). As with cytochrome *b* alone, concatenated haplotype diversity was higher in the west (0.90, SD = 0.017) than east (0.85, SD = 0.027). However, nucleotide diversity was higher in the east (0.0086, SD = 0.00075) than west (0.0058, SD = 0.00023).

**Table 1:** Occurrence of 29 concatenated 785 bp cytochrome *b* and D loop haplotypes among 10 gray fox and 3 island fox sampling locations.

Sample location	*n*	A-4	A-7	A-23	A-5	A-6	B-2	B-3	C-13	C-14
**1—Western Cascades**	14	-	-	-	7	1	-	-	-	-
**2—North Coast**	34	-	-	-	1	-	-	2	-	-
**3—Central coast**	10	-	-	-	-	-	-	-	-	-
**4—Southern California**	2	-	-	-	-	-	-	-	-	-
**5—Western Sierra/San Joaquin Valley**	13	-	-	-	-	-	-	-	5	1
**6—Sacramento Valley**	27	-	-	-	1	-	2	4	5	-
**7—Eastern Sierra Nevada**	5	-	-	-	-	-	-	-	-	-
** Nevada**	1	-	-	-	-	-	-	-	-	-
** Washington**	1	-	-	-	-	-	-	-	1	-
**8—Island foxes**	11	4	5	2	-	-	-	-	-	-
** Santa Cruz Isl.**	4	4	-	-	-	-	-	-	-	-
** San Nicolas Isl.**	5	-	5	-	-	-	-	-	-	-
** San Clemente Isl.**	2	-	-	2	-	-	-	-	-	-
** Georgia**	27	-	-	-	-	-	-	-	-	-
**Total**	145	4	5	2	9	1	2	6	11	1
**Sample location**	C-16	D-2	D-9	D-10	D-11	D-12	D-15	D-16	D-17	E-1
**1—Western Cascades**	-	-	3	-	-	-	3	-	-	-
**2—North Coast**	-	-	21	2	3	4	1	-	-	-
**3—Central coast**	1	-	1	-	-	-	-	6	-	-
**4—Southern California**	-	-	-	-	-	-	-	-	-	-
**5—Western Sierra/San Joaquin Valley**	-	-	-	-	-	-	4	-	-	3
**6—Sacramento Valley**	-	1	5	-	-	1	5	-	-	3
**7—Eastern Sierra Nevada**	-	-	-	-	-	-	-	-	-	-
** Nevada**	-	-	-	-	-	-	-	-	1	-
** Washington**	-	-	-	-	-	-	-	-	-	-
**8—Island foxes**	-	-	-	-	-	-	-	-	-	-
** Santa Cruz Isl.**	-	-	-	-	-	-	-	-	-	-
** San Nicolas Isl.**	-	-	-	-	-	-	-	-	-	-
** San Clemente Isl.**	-	-	-	-	-	-	-	-	-	-
** Georgia**	-	-	-	-	-	-	-	-	-	-
**Total**	1	1	30	3	3	5	13	6	1	6
**Sample location**	J-13	K-15	L-18	M-8	F-21	G-Uci22	H-19	H-20	H-Uci23	I-22
**1—Western Cascades**	-	-	-	-	-	-	-	-	-	-
**2—North Coast**	-	-	-	-	-	-	-	-	-	-
**3—Central coast**	-	1	-	1	-	-	-	-	-	-
**4—Southern California**	-	-	2	-	-	-	-	-	-	-
**5—Western Sierra/San Joaquin Valley**	-	-	-	-	-	-	-	-	-	-
**6—Sacramento Valley**	-	-	-	-	-	-	-	-	-	-
**7—Eastern Sierra Nevada**	5	-	-	-	-	-	-	-	-	-
** Nevada**	-	-	-	-	-	-	-	-	-	-
** Washington**	-	-	-	-	-	-	-	-	-	-
**8—Island foxes**	-	-	-	-	-	-	-	-	-	-
** Santa Cruz Isl.**	-	-	-	-	-	-	-	-	-	-
** San Nicolas Isl.**	-	-	-	-	-	-	-	-	-	-
** San Clemente Isl.**	-	-	-	-	-	-	-	-	-	-
** Georgia**	-	-	-	-	6	2	3	6	5	5
**Total**	5	1	2	1	6	2	3	6	5	5

Haplotype names follow the convention that the cytochrome *b* fragment haplotype is named before the dash (by letter) and the D loop fragment haplotype is named following the dash (e.g., Sacks et al. 2010). All our D loop haplotypes were named with numerals, but the previously published D loop haplotypes use “Uci” in the name in keeping with previously assigned names (Bozarth et al. 2011). Numerals to left of sample names were for mapping purposes.

### Eastern versus western foxes

**Fig 2:**
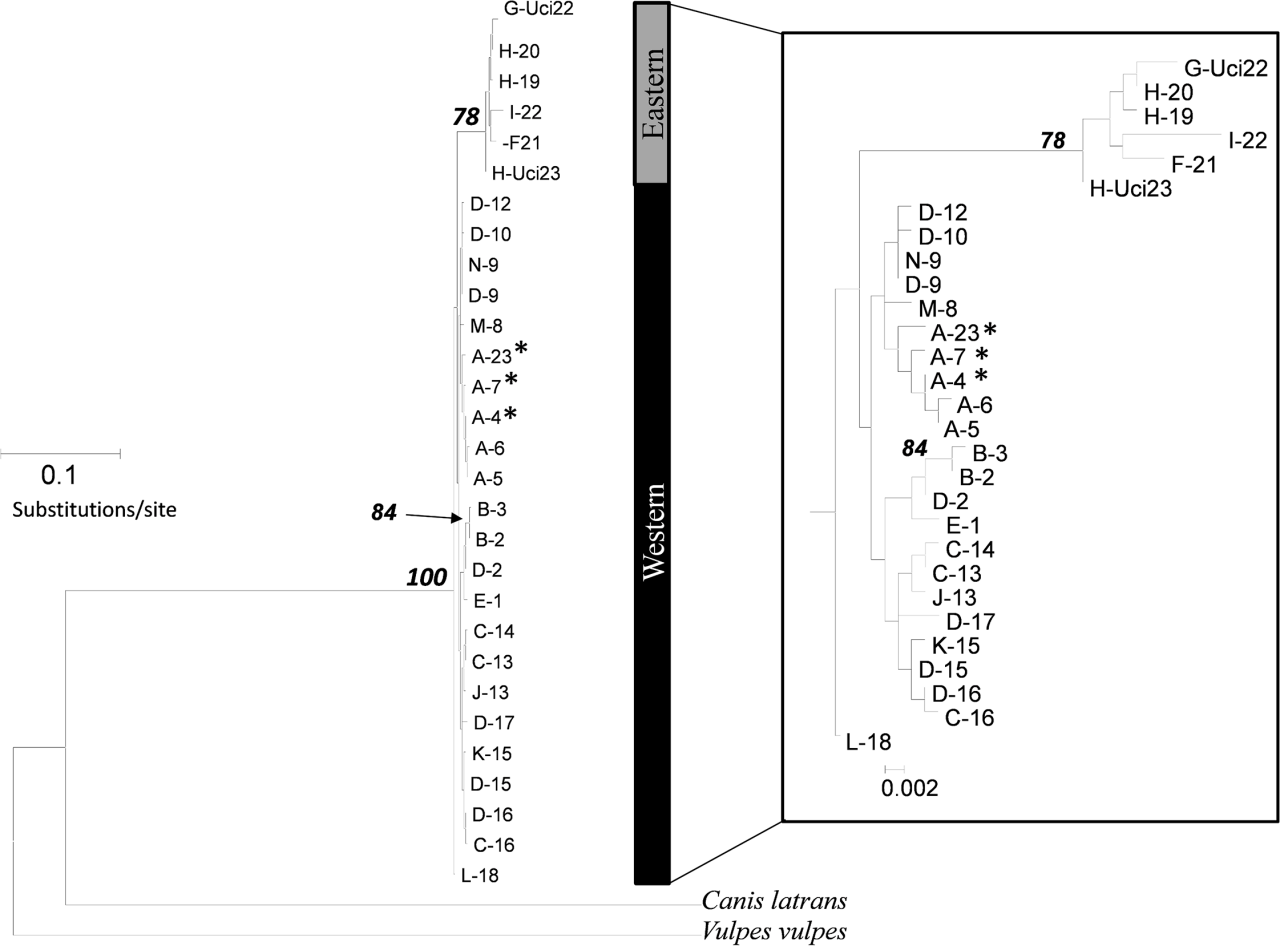
Maximum likelihood tree of concatenated 785 bp cytochrome *b* and D loop sequences. Tree was constructed using a HKY+ Γ model of DNA substitution. Bootstrap values >75 are shown at nodes, although values on inner nodes are shown only on the *Urocyon* clade expansion. Asterisks denote haplotypes of island foxes.

**Fig 3:**
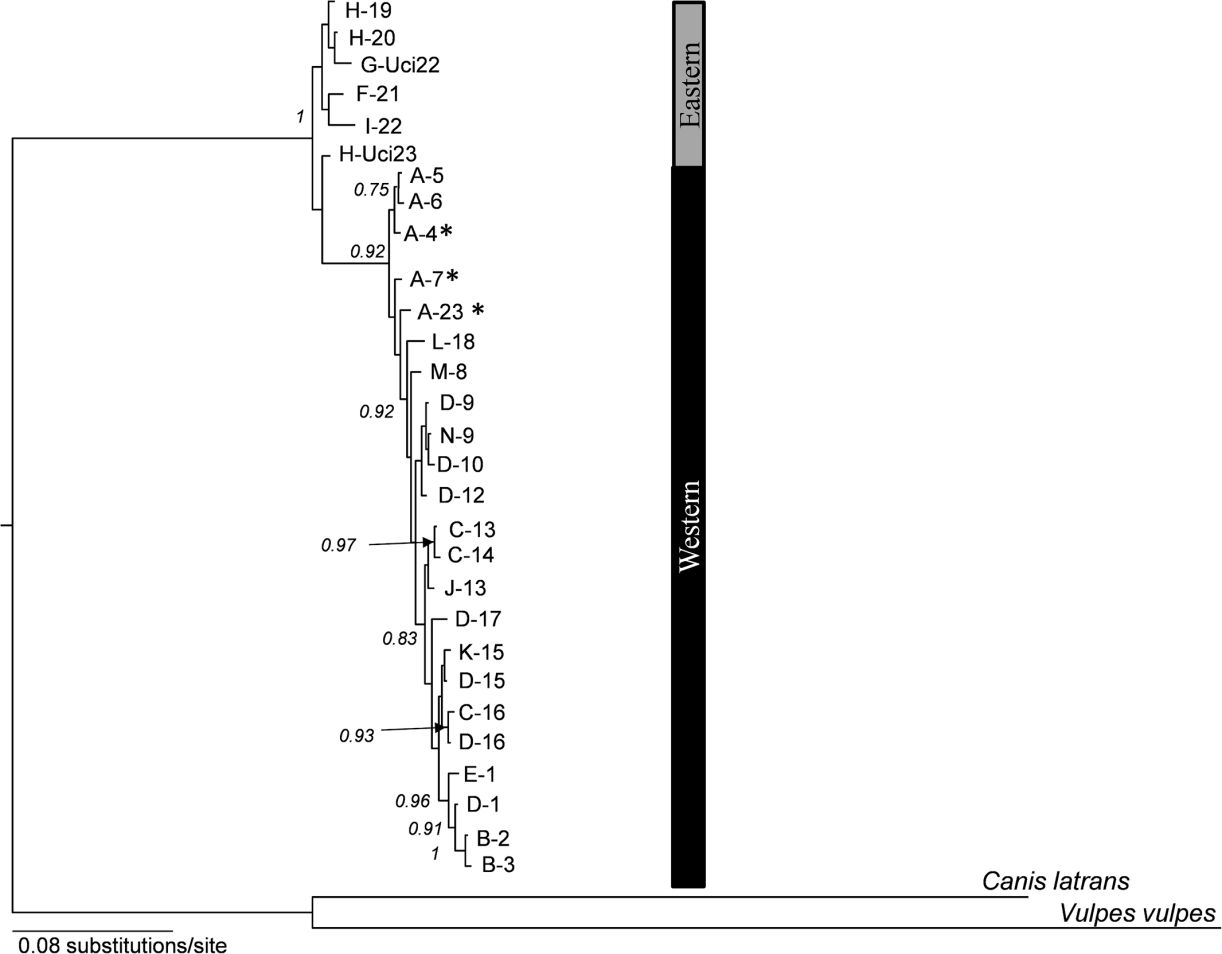
Bayesian phylogeny of concatenated 785 bp cytochrome *b* and D loop haplotypes. Tree was constructed using HKY (cytochrome *b*) and HKY+Γ (D loop) models of DNA substitution. Bayesian posterior probability values >0.75 are shown at nodes. Asterisks denote haplotypes of island foxes.

The maximum likelihood and Bayesian trees were ambiguous with respect to the basal versus derived positioning of putative clades and neither tree had sufficient resolution to evaluate reciprocal monophyly. However, the maximum likelihood tree provided moderate to high bootstrap support (78%) for the monophyly of a clade composed solely of all eastern haplotypes (Fig 2), whereas the Bayesian tree provided a similar degree of support (0.92 Bayesian Posterior Probability) for the monophyly of a clade composed solely of all western fox haplotypes (Fig 3).

The sequence divergence between eastern and western samples (2.93%) minus the average sequence divergence within samples (i.e., nucleotide diversity, 0.72%) yielded a net sequence divergence of 2.2%. However, the cytochrome *b* fragment alone (*n* = 180 sequences) yielded a sequence divergence of 1.3% (adjusted as above for within-population sequence divergence) between western and eastern *Urocyon*. (We also estimated sequence divergence for the concatenated D loop and cytochrome *b* fragments using the Kimura 2 parameter models, but which extended the estimates only slightly, <4%.) If both fragments had accumulated mutations linearly with time since divergence, we would expect sequence divergence in the D loop to be 5 to 7 times that of the cytochrome *b* portion, based on the relative substitution rates of these fragments observed on more recent time frames (e.g., [5,20]). Consistent with this expectation, within the western clade, we observed that nucleotide diversity of the D loop fragment (0.0184) was 6.1 times higher than that in the cytochrome *b* fragment (0.0030). Thus, we conclude that the rapid mutation rate of the D loop over the long time period of interest rendered it an inappropriate marker for assessing divergence between eastern and western *Urocyon*.

**Fig 4:**
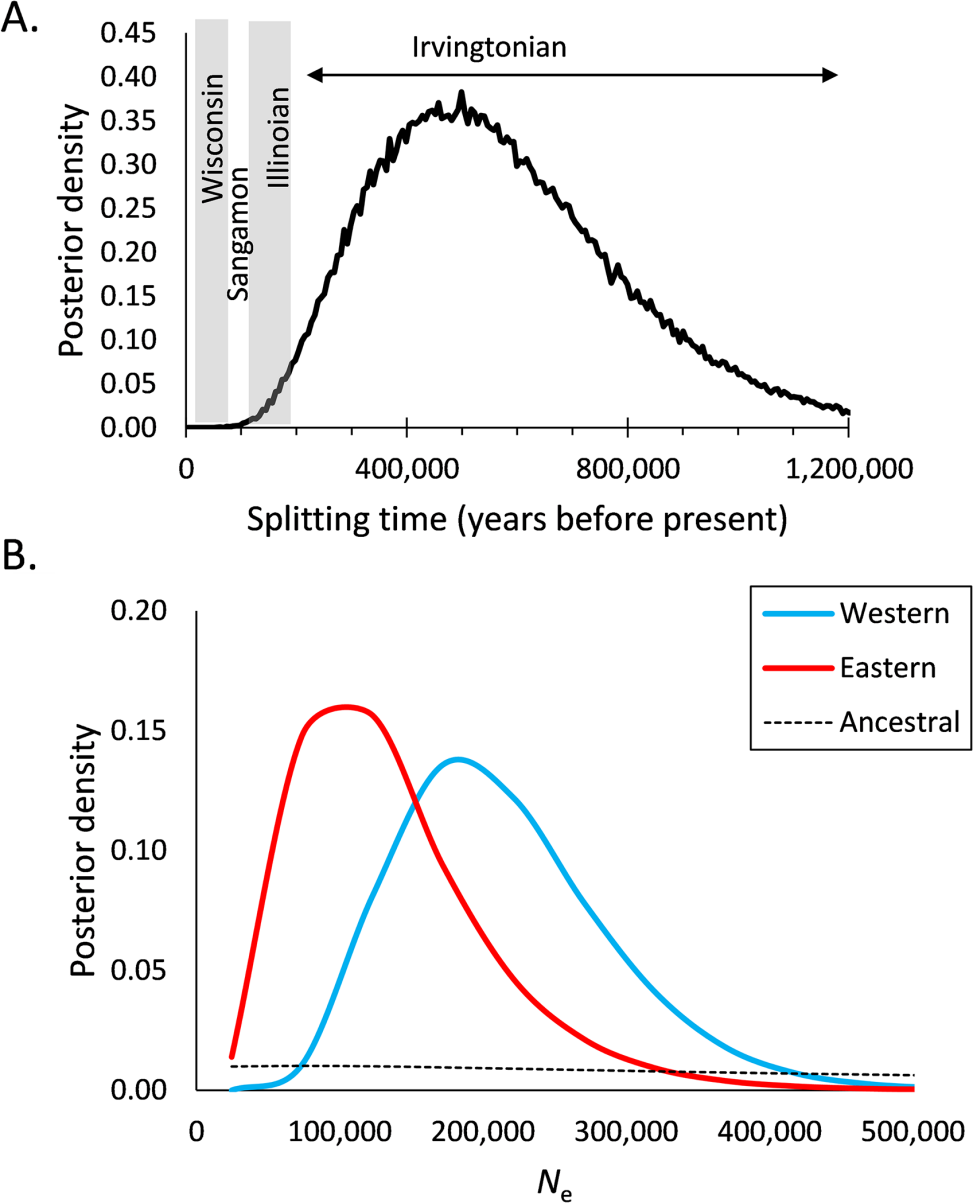
Coalescent analysis of mitochondrial cytochrome *b* sequences of 180 *Urocyon* in IMa2, providing (A) an estimate of splitting time separating eastern and western *Urocyon* and (B) estimates of effective population sizes (*N*_e_) of western, eastern, and ancestral *Urocyon* spp.

The coalescent analysis of the 180 cytochrome *b* sequences in IMa2 indicated estimates of splitting time (mean posterior) between eastern and western *Urocyon* at 575,168 YBP (95% HPD = 174,144–1,041,913) (Fig 4A). The contemporary effective population size of the western *Urocyon* (215,171, 95% HPD = 122,983–368,949) was estimated to be larger than the eastern population (138,085, 95% HPD = 24,597–319,756), although HPD intervals overlapped estimates (Fig 4B). The posterior distribution for the ancestral population size was relatively flat and, therefore, uninformative.

**Fig 5:**
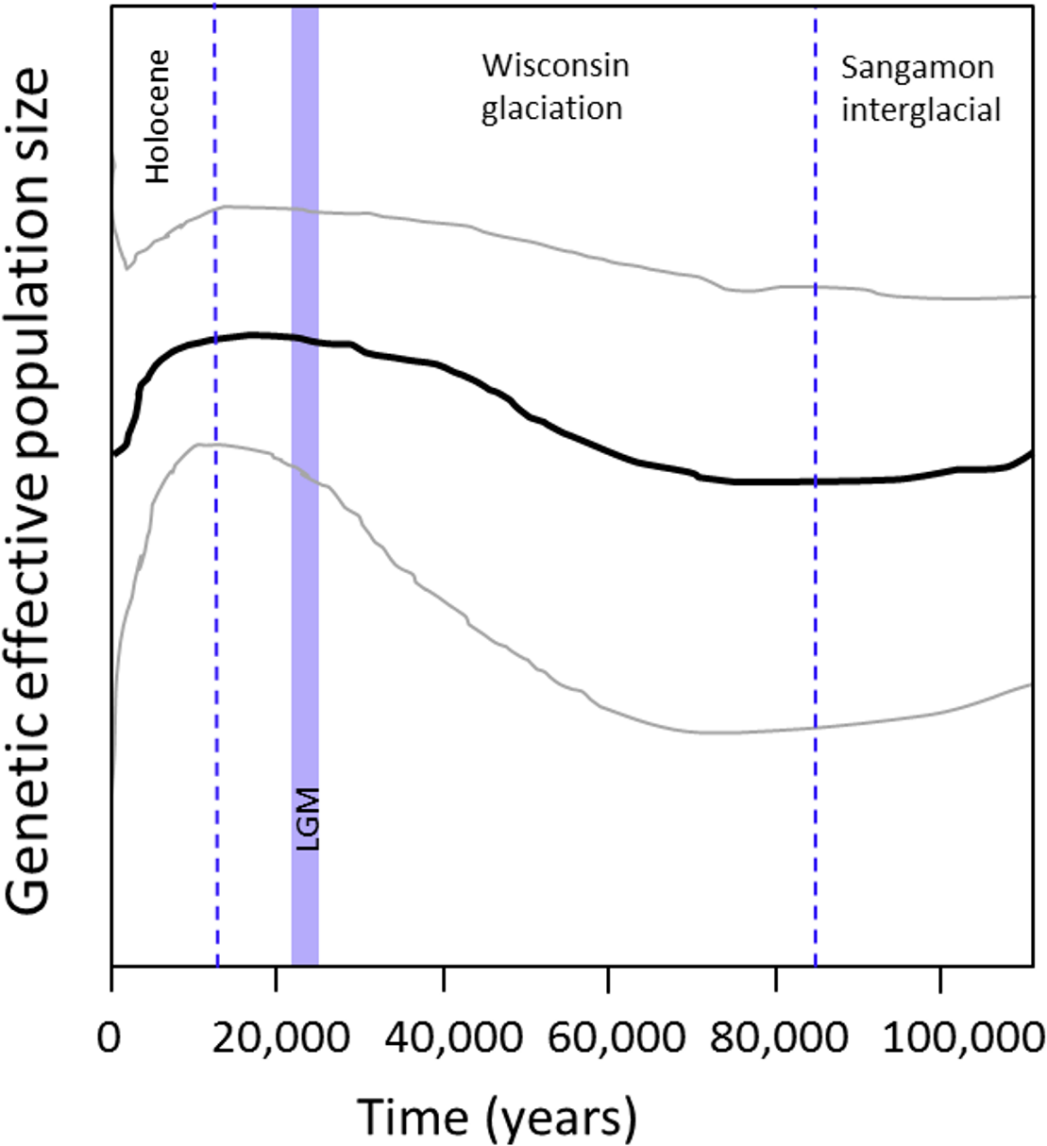
Bayesian skyline plot illustrating Pleistocene increase and Holocene decline in population size of western *Urocyon* based on 785 bp of concatenated cytochrome *b* and D loop sequence data. The black line represents the median population size, while the lines above and below represent the 95% highest posterior density (HPD). Dashed lines indicate glacial-interglacial boundaries and the blue shaded rectangle indicates the last glacial maximum (LGM) for reference to the population sizes. The estimated root height (coalescence time) was 96,860 years (95% HPD: 55,318–144,274 years) or 48,380 generations (assuming a 2-year generation time).

Comparison of Fu’s *F*_S_ statistics to those simulated under constant-population size models (based on the entire concatenated sequences) indicated no statistically significant evidence of recent population expansions in either the (south)eastern (*F*_S_ = 5.07, *P* = 0.96) or the (south)western (*F*_S_ = -4.07, *P* = 0.14) population, consistent with long-term stability (or population declines) in both portions of the continent. A Bayesian skyline plot using the western sample, however, indicated a gradual increase in abundance beginning around 70,000 YBP (35,000 generations), stabilizing long before the last glacial maximum (20,000 YBP), and remaining relatively stable through the first half of the Holocene, but followed by a decline over the past several thousand years (Fig 5). This analysis also generated an age estimate of 96,760 YBP (95% HPD: 55,318–144,274 YBP) for the root height, i.e., coalescence time, of western *Urocyon* sequences.

### Phylogenetic structure of western *Urocyon*

**Fig 6:**
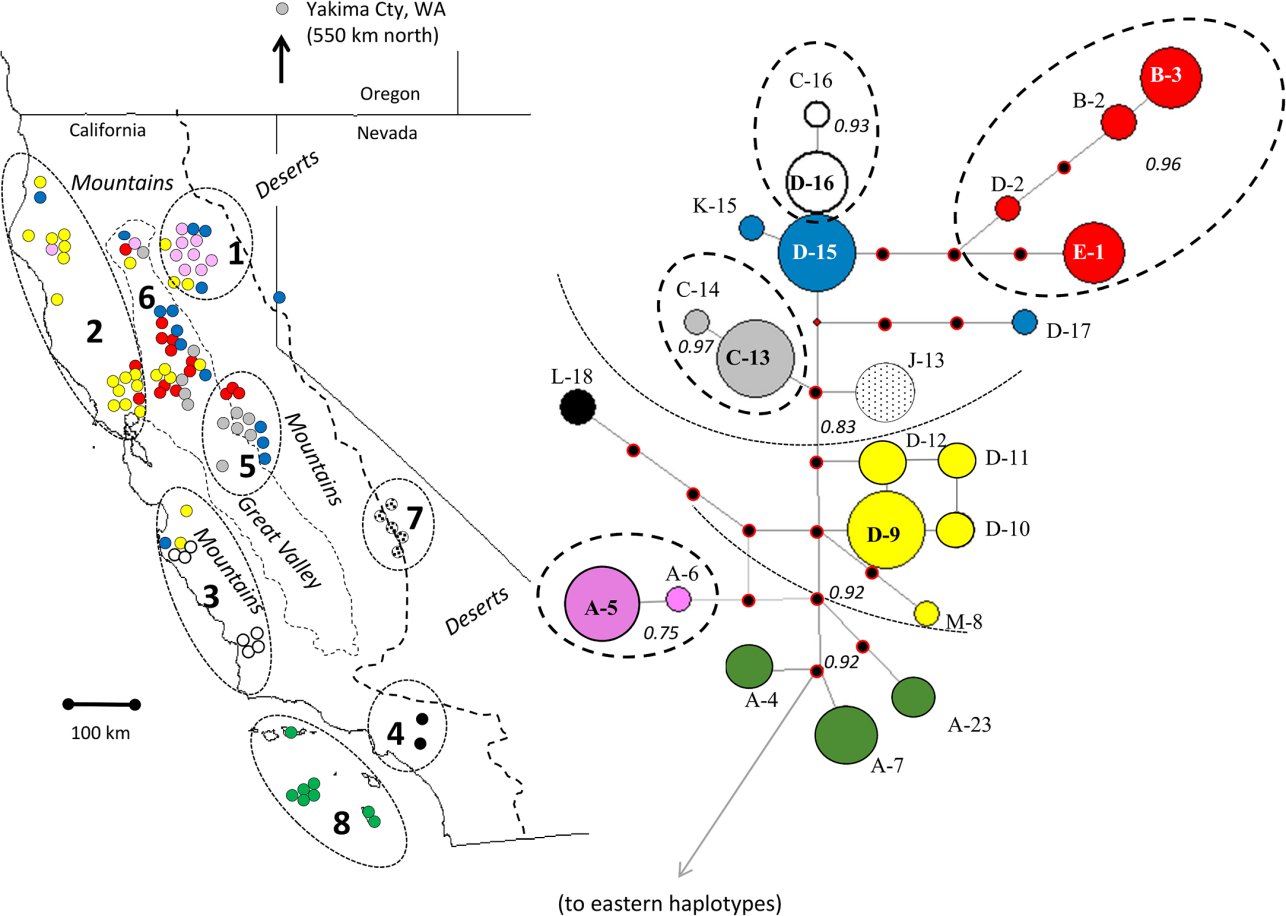
Median-joining network of 785-bp composite cytochrome *b* and D loop haplotypes. Haplogroups are color-coded for reference to map. Numbered samples correspond to those listed in Table 1. Haplotypes in green correspond to island foxes from Santa Cruz Island (A-4), San Nicholas Island (A-7), and San Clemente Island (A-23). All others represent gray foxes from the mainland. Dashed ellipses on network enclose nested tip clades and dotted arcs denote nesting clades according to the Bayesian tree (Fig 3), with Bayesian Posterior Support indicated with numerals in italics. Positioning of the root is in reference to the eastern haplotypes as indicated on the Bayesian tree (Fig 3). Nodes are approximately proportional to samples size for *n* < 10 (i.e., circle size was the same for *n* = 10–30). Inferred, unsampled haplotypes are marked with black and red circles.

Two phylogeographic patterns were evident in the western sample (Fig 6). First, several tip haplogroups (used here to indicate, collectively, closely related haplotypes with unresolved topology and well-supported nested clades) had localized distributions, suggesting that lineages had been geographically stable long enough for mutations to accumulate in those locations without diffusing over long distances (within haplogroups). For example, the “white” haplogroup (as per color-coding in Fig 6) was found solely along the Central Coast and the “red” haplogroup was found primarily in the Sacramento Valley and vicinity. Second, despite the geographic and phylogenetic concordance within haplogroups, there was little phylogenetic concordance among haplogroups. Most notably, the “pink” haplogroup was at opposite end of our study region from the “green” haplogroup (island foxes). Conversely, the distantly related “pink” and “yellow” haplogroups had similar distributions. Moreover, there was a notable lack of interior haplogroups, i.e., many missing links among haplogroups, consistent with antiquity.

The accumulation of substitutions within haplogroups provided an indication of timeframes corresponding both to the local accumulation of mutations in-situ and the age of the entire western lineage as rooted to the eastern lineage (Table 2). The six tip haplogroups ranged in estimated age from ~4,700 to 33,700 generations, translating to 9,400 to 67,400 YBP, with most estimates falling prior to the last glacial maximum. The age estimated for the entire western clade (including island foxes) was ~61,000 generations (within the 95% HPD for root height estimated from the Bayesian skyline analysis above), with successively nested subsets of this clade estimated similarly at 46,000 to 50,000 generations, all corresponding approximately to the Sangamon interglacial period (125,000–85,000 YBP).

**Table 2:** Average (and standard deviation, SD) numbers of mutations separating descendent haplotypes from ancestral nodes (rho) and corresponding estimates of time to most recent common ancestor (TMRCA) assuming an average substitution rate of 10.8% per million generations in the 785 bp concatenated cytochrome *b* and D loop fragment.

Ancestral node^1^	Descendent haplotypes	Rho estimate (SD)	TMRCA (SD) in generations	Colors in network^2^
**Basal MV**	Island fox haplotypes (A-4, A-7, A-23)	1.5 (0.729)	17,625 (8,566)	Green
**Basal MV**	All western *Urocyon* (A-4, A-5, A-6, A-7, A-23, B-2, B-3, C-13, C-14, C-16, D-2, D-9, D-10, D-12, D-15, D-16, D-17, E-1, J-13, K-15, L-18, M-8, N-9)	5.22 (1.538)	61,335 (18,072)	All
**Interior MV**	B-2, B-3, C-13, C-14, C-16, D-2, D-9, D-10, D-12, D-15, D-16, D-17, E-1, J-13, K-15, L-18, M-8, N-9	3.95 (1.237)	46,413 (14,535)	Yellow, black, gray, stippled, blue, white, red
**Interior MV**	B-2, B-3, C-13, C-14, C-16, D-2, D-15, D-16, D-17, E-1, J-13, K-15	4.26 (1.5)	50,055 (17,625)	gray, stippled, blue, white, red
**Interior MV**	D-9, D-10, D-12, M-8	1.2 (0.968)	14,100 (11374)	Yellow
**Interior MV**	C-13, C-14	1.08 (1.003)	12,725 (11,785)	Gray
**D-15**	D-16, C-16	0.4 (0.354)	4,700 (4,160)	White
**A-4**	A-5, A-6	1 (0.914)	11,750 (10,740)	Pink
**Interior MV**	B-2, B-3, D-2, E-1	2.87 (1.187)	33,723 (13,947)	Red

^**1**^Ancestral nodes for rooting were median vectors (MV) corresponding to the basal node identified in the Bayesian tree (Fig 3) or the one immediately interior to the descendent haplotypes, or were the sampled haplotype immediately interior to the descendent haplotypes.

^**2**^Colors correspond to those in Fig 6.

Lastly, we wished to preliminarily investigate the geographic patterns of connectivity among sampling locations. Assuming one split among western sampling locations (i.e., *K* = 2), the SAMOVA identified the Eastern Sierra Nevada sampling location as distinct from all other western locations, including Island foxes, although the model was not statistically significant (Table 3). The lowest number of groupings that was statistically significant was *K* = 3, which split out both the Eastern Sierra Nevada sample and island foxes as distinct groups relative to other western gray fox sampling locations. Although Φ_CT_ also were statistically significant for *K* = 4–6 groupings, their values did not increase substantially with higher *K* nor were models nested hierarchically. A Mantel test based on the five California sample sites that clustered together at *K* = 3 in the SAMOVA indicated a significant pattern of isolation by distance (Mantels *r* = 0.62, *P* = 0.034).

**Table 3:** Best population groupings for *K* = 2–6 for combined cytochrome *b* and D loop sequences and statistical results from SAMOVA.

Sampling location	*K* = 2	*K* = 3	*K* = 4	*K* = 5	*K* = 6
**Western Cascades**	A	A	C	E	E
**North Coast**	A	A	D	D	D
**Central Coast**	A	A	A	A	F
**Western Sierra/San Joaquin Valley**	A	A	A	A	A
**Sacramento Valley**	A	A	A	A	A
**Eastern Sierra Nevada**	B	B	B	B	B
**Island foxes**	A	C	C	C	C
**Φ**_**CT**_	0.21	0.22	0.20	0.23	0.26
***P***	0.13	0.05	0.01	0.02	0.05

Combining our D loop sequences (406 bp) with those of Bozarth et al. [20], we constructed an unrooted tree including all known D loop haplotypes, which strongly supported the monophyly of an eastern clade (99% bootstrap support) relative to a western clade (S1 Fig). We then compared statistics of our 406 bp D loop dataset to the corresponding data set of Bozarth et al [20]. Western gray foxes (i.e., mainland) exhibited higher haplotype diversity than Island foxes or northeastern gray foxes, both founded during the Holocene, and similar haplotype diversity to the older (refugial) southeastern gray foxes (Table 4). Similarly, breaking up the western mainland sample according to locality resulted in consistently higher haplotype diversity estimates than were observed in the northeastern US. Coastal sites tended to have lower diversity than inland sites but there was no latitudinal pattern. Indeed, the highest haplotype diversity estimate was in the Sacramento Valley in northern California. Nucleotide diversity estimates were more variable, but nevertheless showed similar values between our large samples of western mainland gray foxes (this study) and southeastern gray foxes [20]. Similarly to the Southeast sample of Bozarth et al. [20], none of our samples had statistically significant Fu’s *F*_s_ estimates.

**Table 4:** Estimates of haplotype and nucleotide diversity in western gray foxes (mainland), island foxes, and eastern gray foxes from the present study, compared to those from Bozarth et al. (2011) for reference using 406 bp of D loop sequence common to both studies.

Sample	Subsample	*n*	Putative age	Haplotype diversity (SD)	Nucleotide diversity (SD)	Reference
**Island foxes**		11	Holocene	0.691 (0.086)	0.049 (0.0011)	This study
**Western**		107	Pleistocene	0.865 (0.019)	0.0083 (0.0003)	This study
	W. Cascades	(14)		0.703 (0.095)	0.0057 (0.0010)	This study
	North Coast	(34)		0.606 (0.091)	0.0036 (0.0010)	This study
	Central Coast	(10)		0.644 (0.152)	0.0180 (0.0046)	This study
	W. Sierra/SJV	(13)		0.756 (0.070)	0.0110 (0.0047)	This study
	Sacramento V	(27)		0.892 (0.025)	0.0085 (0.0004)	This study
**Southeast**		27	Pleistocene	0.846 (0.027)	0.0134 (0.0010)	This study
**Southeast**		158	Pleistocene	0.887	0.0099	Bozarth
**Northeast**		71	Holocene	0.527	0.0020	Bozarth

Local estimates are also provided for 5 sampling locations within the western mainland population.

Comparison of our sequences to those of Hofman et al. [17] indicated most major western *Urocyon* clades and haplogroups, and internal haplotypes in particular, were represented in both datasets (S2 Fig). An exception was the “red” haplogroup of the present study (Fig 6), which was not represented in the Hofman et al. [17] sample. The much larger sample of island foxes by Hofman et al. [17] increased the number of concatenated (785 bp) haplotypes from the three we observed in our sample to a total of six in the combined sample. Thus, it appears that the sampling schemes of the two studies, one of which emphasized island foxes and the other, mainland gray foxes, were both adequate to capture the majority of the basal mitochondrial diversity of western *Urocyon*. The Bayesian skyline plot based on the 25 western gray fox whole mitogenomes [17] showed the same Holocene decline, but not the earlier expansion, as our larger sample of 785 bp sequences (S3 Fig, Fig 5).

Computation of rho estimates from a network constructed from the whole mitogenomes of Hofman et al. [17] indicated an average of 37.04 mutations (i.e., rho = 37.04, SD = 4.09) between ancestral and descendent haplotypes within the western gray fox clade (excluding island fox haplotypes). Applying the same mutation rates of coding and noncoding DNA as we assumed for those portions of the 785 bp of sequence implies an age of approximately 60,000 generations or 120,000 years for the western gray fox clade, in agreement with inferences from our data. Specifically, we assumed 2.8% per million generations in the coding DNA (in this case, 15,478 bp) and 17.75% per million generations in the D loop portion (in this case, 982 bp), i.e., averaging 3.7% per million generations or 1.85% per MY in the entire mitogenome (e.g., as estimated for other canids [2,8]). Applying the same mutation rates to the island fox clade, along with two standard deviations to either side produced an estimated time to most recent common ancestor of approximately 35,000 to 67,000 years for the entire island fox clade and 7,300 to 19,700 years for the subclade associated with the northern islands (S4 Fig). The subclade associated with the southern islands, however, was a similar age as the entire island fox clade, ranging 25,400 to 56,600 years since descending from a common ancestor (S4 Fig). Even the clade nested within this one corresponding to two of the southern islands was estimated to be 9,000 to 35,000 years old.

## Discussion

### Divergence between eastern and western *Urocyon*

Our results demonstrate that gray foxes in the eastern and western United States were highly divergent. First, our estimate based on the coalescent simulations performed in IMa2 indicated an estimated split time of 575,168 YBP (95% HPD = 1,041,913–174,144 YBP), which provided robust statistical support for the general timeframe. The simplest measure of this split, however, was in terms of the sequence divergence between eastern and western *Urocyon* in the cytochrome *b* fragment. This sequence divergence can be compared directly to interspecific branching points among other foxlike canids. In particular, the 1.3% cytochrome *b* divergence we observed between eastern and western *Urocyon* was >162% that observed between kit and swift foxes (0.8%), 72% as much as that between the latter two and arctic foxes (*Vulpes lagopus*; 1.8%), and 15% that between these three foxes and red foxes [2,8]. The branching points for these taxa have been independently calibrated to nuclear DNA and fossil evidence or more extensive mitochondrial DNA [2,8,10], indicating divergence times of 0.5 MY, 0.8 MY, and 2.9 MY, respectively, translating to a 1.6 to 3% sequence divergence per MY (see also [46]). Using this range to bracket cytochrome *b* sequence divergence between eastern and western gray foxes implies divergence dating from 813,000 to 433,000 YBP, consistent with our IMa2 estimate. Thus, it appears that eastern and western *Urocyon* trace their divergence back to the late Irvingtonian (Middle Pleistocene), and well within the range of distinct sister taxa.

The fossil record is poor for *Urocyon* prior to the Wisconsin glaciation, but the earliest specimens identified as *U*. *cinereoargenteus* were from Arkansas and further east [11]. However, nominal *Urocyon galushai* were identified in Irvingtonian sites in southern California and *U*. *citrinus* from the Irvingtonian in Florida [47]. During the Sangamon interglacial (~125,000–85,000 YBP), *U*. *cinereoargenteus* specimens were known from Florida and Mexico. During the Wisconsin glaciation (85,000–11,000 YBP), fossils designated *U*. *cinereoargenteus* were distributed essentially throughout the southern portion of the current range, including California, Arizona, New Mexico in the west and Florida, Georgia, and Pennsylvania (northern extent) in the east [11]. Thus, one possibility is that *U*. *cinereoargenteus* expanding from Mexico replaced *U*. *galushai* in the west and *U*. *citrinus* in the east during the late Irvingonian or early Wisconsin. Alternatively, in light of our genetic findings, which put the split between contemporary eastern and western *Urocyon* back to the Irvingtonian, it seems plausible that contemporary western *U*. *cinereoargenteus* stems from the previously identified Irvingtonian species, *U*. *galushai* and *U*. *citrinus*, respectively.

The prospect of such deep phylogenetic divergence between western and eastern *Urocyon* is complemented by the possibility of adaptive differentiation associated with evolution in distinct environments. Today, *Urocyon* of the east are associated closely with mesic hardwood forests, whereas those of California are primarily associated with arid scrub habitats [12]. In general, the southeast and California bioregions were composed similarly to the present during the late Pleistocene [11]. Nuclear genetic data are needed to confirm the divergence observed in the mitochondrial gene tree between of eastern and western *Urocyon* and to assess population history as evidenced throughout the entire genome. Samples from intervening geographical regions as well as further south are also needed to assess gene flow, discrete breaks, and relationships to *Urocyon* in Mexico, Central America, and South America.

### Antiquity of gray and island foxes in California

Related to the splitting time between eastern and western *Urocyon* was the age of *Urocyon* in the western portion of its range and the divergence time between extant (or at least sampled) gray and island fox matrilines. Our estimates of these timeframes based both on our data set and reanalysis of that of Hofman et al. [17] were in close agreement. Specifically, we estimated the coalescence time for western *Urocyon* to be on the order of 100,000 YBP or more and estimated the time to most recent common ancestors of the island fox clade at 35,000 to 51,000 YBP. Additionally, the topology based on whole mitogenomes (e.g., Figs 2 and 3 in [17]; S4 Fig) implies a similar timeframe (35,000 to 51,000 YBP) for the divergence of all extant island fox matrilines from all gray fox matrilines, including the most phylogenetically proximate gray fox haplotypes sampled from northern California ([17], this study), placing this event prior to the last glacial maximum.

However, these timeframes were significantly deeper than the corresponding estimates by Hofman et al. [17], which, in turn, was due to differences in assumptions about mitochondrial mutation rates in *Urocyon*. Whereas we assumed that substitution rates (and generation times) of *Urocyon* were similar to those of other canids (and substitution rates appear to be similar throughout the Carnivora [46]), Hofman et al. [17] assumed that the most recent common ancestor to extant island foxes coincided closely (~9,200 years ago) with the first known occurrence of foxes on the islands 7,100 years ago (as independently determined via radiocarbon-dated specimens). The latter assumption seems unlikely to us based on the shapes of the island fox networks, both from whole mitochondrial sequences [17] and our concatenated sequences (Fig 6, S4 Fig). In general, networks were more reticulated than star-shaped (as would be expected if these foxes arose from a recent common ancestral matriline), and had multiple missing links, which also seems implausible on such a short timeframe (despite population fluctuations due to epizootics, etc.). These networks therefore seem inconsistent with expansion from a single founder (see, for example, patterns in Australian dingoes, which arose ~8,000 to 5,000 years ago [48,49]).

Given that island foxes apparently did not arrive on the Channel Islands until 9,200 to 7,100 years ago (reviewed by [17,50]), we suggest that contemporary island foxes must trace to a minimum of three or four distinct founder matrilines. One founder associated with the northern islands potentially rooted the corresponding subclade, which did appear star-shaped (see Fig 2 in [17]) and which we dated at 7,300 to 19,700 YBP (S4 Fig). Another founder likely corresponded to the highly divergent haplotype cluster unique to San Clemente Island, and one or two founders potentially explain the haplotypes currently observed on San Nicolas and Santa Catalina Islands (excluding the haplotype also found on Santa Cruz Island). Whether multiple founders were used to start a single founding population (i.e., on one of the islands) that subsequently gave rise to populations on other islands or as independent mainland founders of distinct island populations cannot be inferred from mitochondrial DNA alone.

Regardless, however, the question remains: What happened to the immediate, mainland maternal ancestors of island foxes in the time since the ancestral island foxes were brought to the islands? Because all island fox matrilines derive from this monophyletic clade, we agree with Hofman et al. [17] that the apparent disappearance of island fox-like haplotypes from the adjacent mainland must reflect events of the Holocene. One possibility is that the clade was present but rare in the southern California mainland and, consequently, not sampled there. Alternatively, island foxes might never have occurred in the Southern and Central California mainland, but, rather, could have been imported from a distant location such as northern California or Mexico. Barring these scenarios, the descendants either died out or, as hypothesized by Hofman et al. [17], moved to the north before being replaced. The late Holocene population decline indicated by our Bayesian skyline analyses supports one of the former possibilities (that the lineages were reduced in frequency or lost due to drift). As discussed below, it seems unlikely that the island fox-like haplotypes (A-5, A-6) found today in the north signify a northern range shift by these ancestors.

### Northern California gray foxes: Recent expansion or ancient roots?

Up to now, we have discussed our age estimates for the coalescence of California and Island fox groups and interpreted our findings to show clear support for a generally ancient age and, therefore, supporting the hypothesis that California as a whole served as a Pleistocene refugium for *Urocyon*. However, we also wished to assess whether northern California, in particular, was part of this refugium (e.g., [15]) or, as hypothesized by Hofman et al.[17], the recipient of a Holocene range expansion from southern California. Notwithstanding the presence of the “island fox-like” haplotypes in northern California, our findings provided no meaningful evidence to support the Holocene range expansion hypothesis in northern California. On the contrary, our findings were consistent with a long-standing, stable population in the area. For example, foxes had high haplotype diversity that was regionally phylogeographically structured and the Bayesian skyline plot showed evidence of a Holocene decline rather than increase. The rho estimates further indicated ancient ages of multiple haplogroups composed of localized haplotypes. The pattern as a whole was in stark contrast to that observed in the northeastern U.S., where gray foxes across a broad area shared primarily two haplotypes, which also were found within the much higher diversity southeast [20]. Gray fox faunal remains were identified in two Wisconsin-age sites from the mountains south of Mount Shasta, Samwell Cave, Potter Creek Cave, confirming the presence of gray foxes in northern California during the late Pleistocene [51]. Conversely, as discussed above, the “island fox-like” haplotypes found in northern California apparently diverged from the actual island fox haplotypes prior to the last glacial maximum.

The landscape of California also could be very important in shaping patterns of migration more so than simple geographic distance. For example, in coyotes, there is high gene flow from north to south along the Pacific Crest and down to the Tehachapi Mountains and the transverse ranges [15]. If gray foxes exhibit a similar pattern, the ecological distance between Mount Lassen and the mountains most proximate to the Channel Islands could be considerably less than that suggested by their Euclidean separation distance. Thus, future analyses of more foxes and using finer resolution markers such as microsatellites can potentially shed further light on the historical and contemporary population genetic structure of gray foxes in California.

## Supporting Information

S1 Fig Unrooted maximum likelihood tree of all 56 D loop haplotypes in this study and that of Bozarth et al. [20]. Topology and branch lengths based on the Kimura 2-Parameter model (+Γ) with bootstrap support based on 500 replicates using 56 distinct D loop haplotypes (406 bp) from the present study and from Bozarth et al. [20]. Haplotypes described for the first time from the present study were indicated by a numeral only; those found by Bozarth are indicated, and those found in both studies are marked with (*). Tree was based on 395 sites that had no deletions in any haplotypes (i.e., only substitutions).
(TIF)

S2 Fig Median-joining network of 785-bp composite cytochrome *b* and D loop haplotypes from Hofman et al. [17] superimposed on the network structure from the present study. Inset shows magnified copy of island fox haplotypes color-coded in terms of island where sampled as per the adjacent legend. Nodes are approximately proportional to samples size in both figures. GenBank accession numbers for Hofman et al. [17] haplotypes were KP128924- KP129108.
(TIF)

S3 Fig Bayesian skyline plot of 27 western gray fox whole mitogenomes from Hofman et al. [17], illustrating mid- to late-Holocene decline. The black line represents the median population size, while the lines above and below represent the 95% highest posterior density (HPD). Dashed lines indicate glacial-interglacial boundaries and the blue shaded rectangle indicates the last glacial maximum (LGM) for reference to the population sizes.
(TIF)

S4 Fig Reconstruction of the network of Hofman et al. [17] from their 185 western *Urocyon* whole mitogenomes (GenBank accession Nos. KP128924-KP129108), in particular illustrating 159 island fox haplotypes and associated rho estimates (and standard deviations). Rho estimates were translated to ages in years assuming a 2-year generation time and substitution rates of 2.8% per million generations in coding DNA and 17.75% per million generations in noncoding DNA (see main text for additional explanation). The small white node corresponds to gray foxes from northern California.
(TIF)
