# Supplementary figures and images for: Mitochondrial Analysis of the Most Basal Canid Reveals Deep Divergence between Eastern and Western North American Gray Foxes (Urocyon spp.) and Ancient Roots in Pleistocene California
Source: PLoS One. 2015 Aug 19;10(8):e0136329. doi: 10.1371/journal.pone.0136329 (PMC4546004; doi:10.1371/journal.pone.0136329)

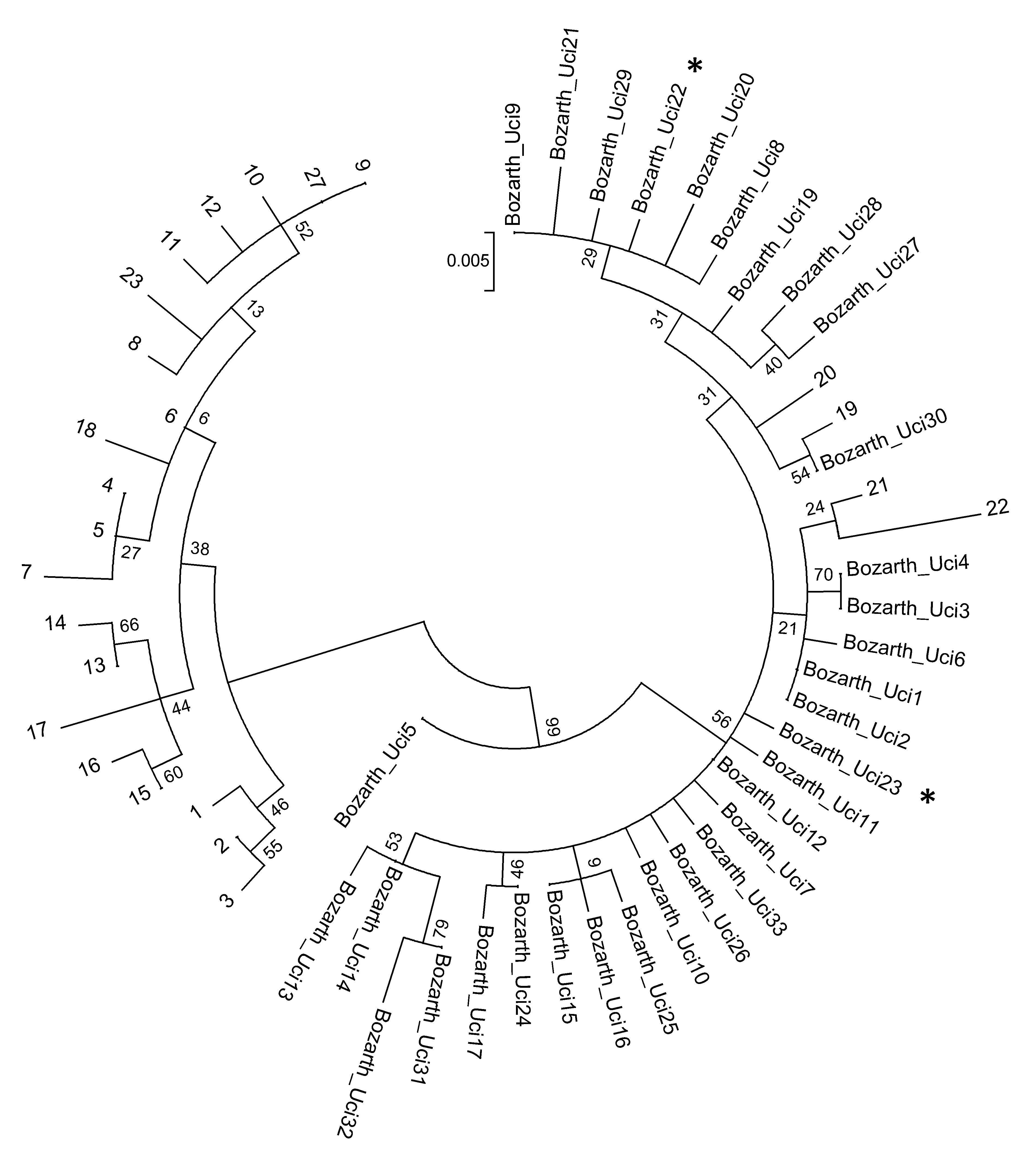

Supplement: S1 Fig — Topology and branch lengths based on the Kimura 2-Parameter model (+Γ) with bootstrap support based on 500 replicates using 56 distinct D loop haplotypes (406 bp) from the present study and from Bozarth et al. [20]. Haplotypes described for the first time from the present study were indicated by a numeral only; those found by Bozarth are indicated, and those found in both studies are marked with (*). Tree was based on 395 sites that had no deletions in any haplotypes (i.e., only substitutions). (TIF) [file pone.0136329.s001.tif]

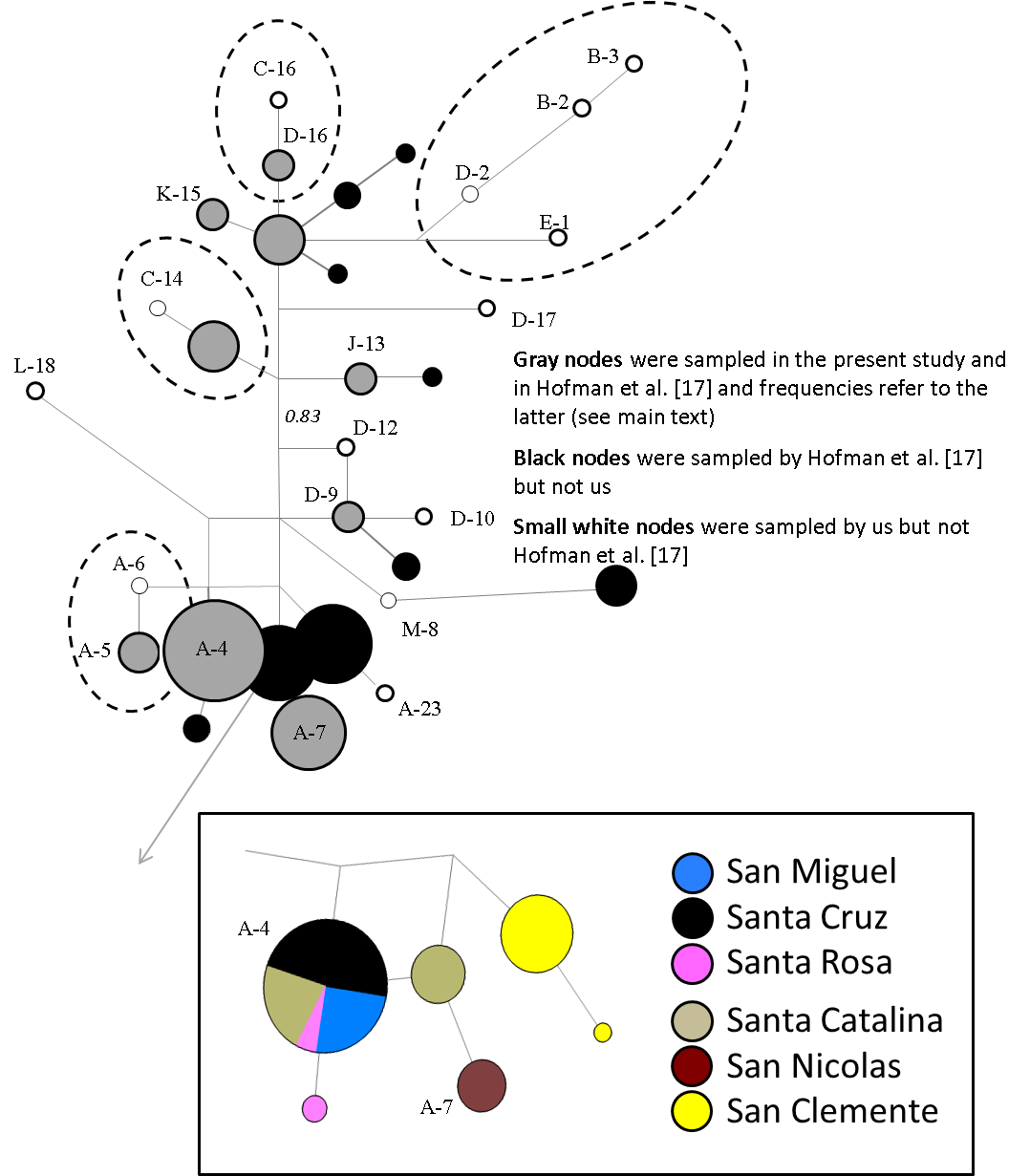

Supplement: S2 Fig — Inset shows magnified copy of island fox haplotypes color-coded in terms of island where sampled as per the adjacent legend. Nodes are approximately proportional to samples size in both figures. GenBank accession numbers for Hofman et al. [17] haplotypes were KP128924- KP129108. (TIF) [file pone.0136329.s002.tif]

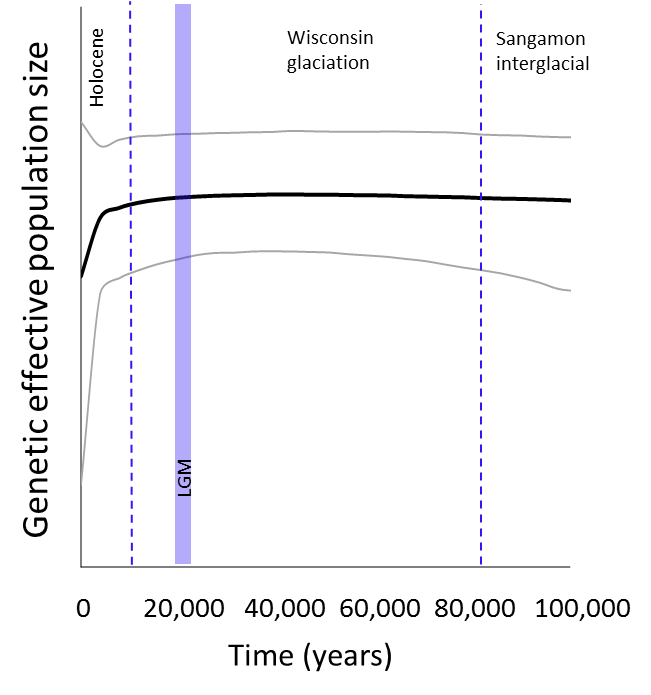

Supplement: S3 Fig — The black line represents the median population size, while the lines above and below represent the 95% highest posterior density (HPD). Dashed lines indicate glacial-interglacial boundaries and the blue shaded rectangle indicates the last glacial maximum (LGM) for reference to the population sizes. (TIF) [file pone.0136329.s003.tif]

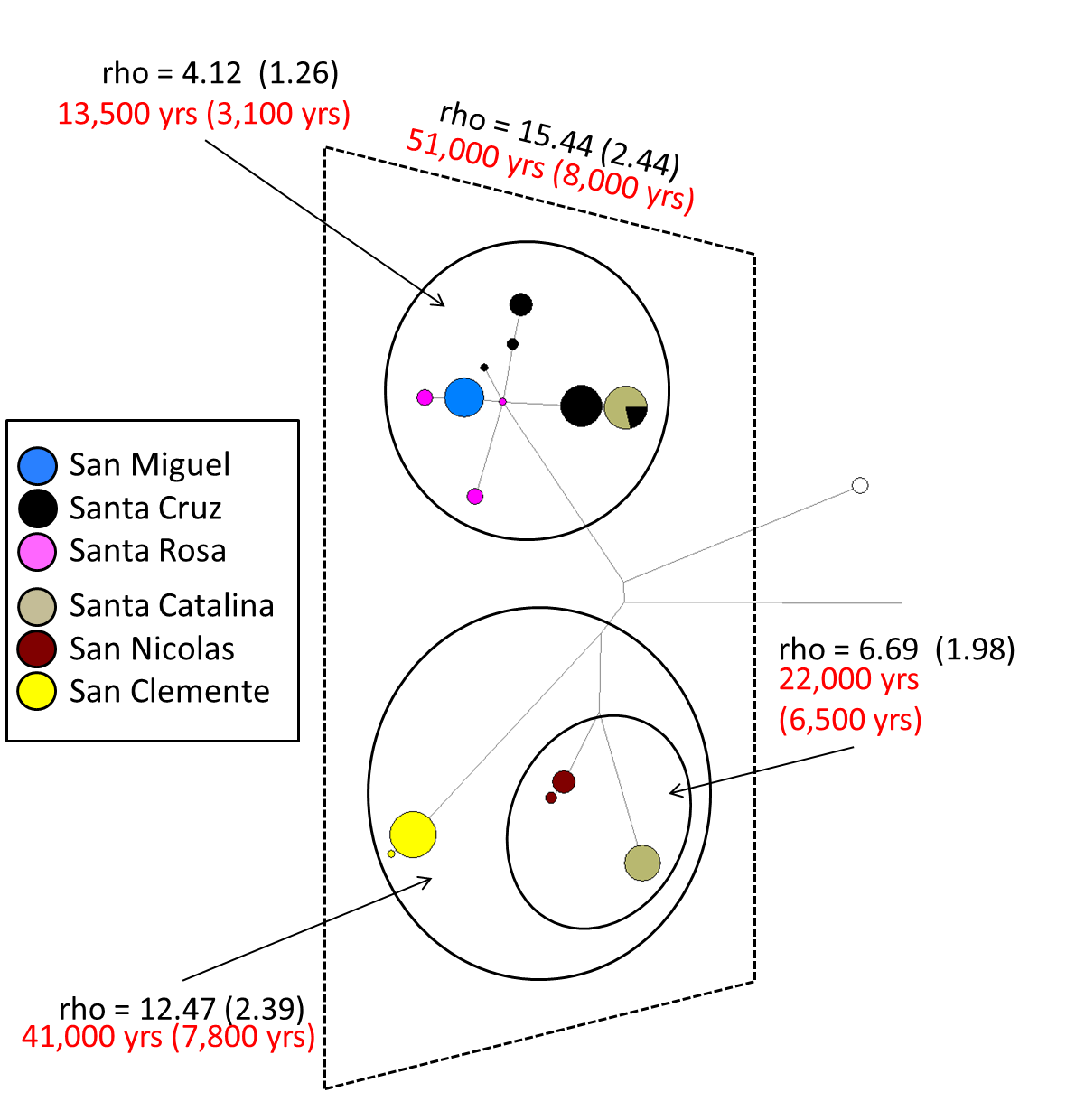

Supplement: S4 Fig — Rho estimates were translated to ages in years assuming a 2-year generation time and substitution rates of 2.8% per million generations in coding DNA and 17.75% per million generations in noncoding DNA (see main text for additional explanation). The small white node corresponds to gray foxes from northern California. (TIF) [file pone.0136329.s004.tif]
